# Dermatologic priorities and considerations when caring for military service members on a global scale

**DOI:** 10.1016/j.jdin.2024.08.007

**Published:** 2024-09-07

**Authors:** Samir Kamat, Michael Auten, Angela Crotty, Curtis L. Hardy, Vikas Shrivastava, Chad Hivnor

**Affiliations:** a Department of Dermatology, Naval Medical Center, San Diego, California; b Department of Medical Education, Icahn School of Medicine at Mount Sinai, New York, New York; c Department of Dermatology, US Naval Hospital, Okinawa, Japan; d South Texas Veterans Health Care System, US Department of Veterans Affairs, San Antonio, Texas

**Keywords:** health care delivery, military dermatology, teledermatology, teledermatoscopy

*To the Editor:* The global military patient population extends into tens of millions and is cared for by military and civilian dermatologists. This article aims to briefly highlight thematic considerations on the care of military service members, an underrepresented population in the global dermatology literature.

Dermatologic presentations among service members remain highly varied, particularly as this population encounters diverse and unique occupational and geographic exposures and may be divided between deployed and nondeployed areas (Table I).^1^ In deployed areas, dermatologic presentation skews toward dermatitis and infectious etiologies, which is not unexpected given service members are likely in close quarters living arrangements and austere geographies.^1^ At the same time, clinicians must be cautious about maintaining a broad differential to include cutaneous oncology, as skin cancers—including common nonmelanoma cancers (eg, basal cell/squamous carcinomas) and rarer presentations (mycosis fungoides)—accounted for 8% of the total at a Combat Dermatology Clinic in Ibn Sina, Iraq, between January 15 and July 15, 2008.^2^

**Table I:** Top dermatologic disease categories in global deployed settings, 2000 to 2022^1^

Country	Deployed location	Length of time and number of dermatology diagnoses	Top 3 disease categories
United States	Iraq	January 2005 to January 2009; *n* = 2197	- Dermatitis and eczematous conditions (13%) - Fungal infections (7%) - Bacterial infection (7%)
United States	Iraq	January 15 to July 15, 2008; *n* = 2696	- Dermatitis and eczematous conditions (18%) - Skin cancer (8%) - Acne (9%)
United Nations	Darfur, Sudan	March 2014 to February 2015; *n* = 542	- Dermatitis and eczematous conditions (38.7%) - Fungal infections (22.5%) - Acne (10.7%)
United Nations	Lebanon	January 2018 to May 2019; *n* = 549	- Dermatitis and eczematous conditions (27.1%) - Fungal infections (13.3%) - Bacterial infections (9.3%)
United States	Composite: Afghanistan, Iraq, Middle East, Africa, Asia	2008-2015; *n* = 429,837	- Fungal infections (11.6%) - Dermatitis and eczematous conditions (8.5%) - Diseases of hair and hair follicles (8.1%)
Britain	Afghanistan	July 2010; *n* = 365	- Fungal infections (25%) - Bacterial infections (12%) - Viral infection (8%)
Korean	Nondeployed	April to September 2010; *n* = 1081	- Acne (43.7%) - Fungal infection (24.5%) - Viral infection (5.7%)
Singaporean	Nondeployed	2007-2009; *n* = 9176	- Fungal infection (28%) - Dermatitis and eczema (24%) - Insect bite reactions (7%)

Beyond the unique epidemiology of dermatologic presentations among service members, treatment decisions must typically consider a patient’s condition and military occupation. The military focuses on mission readiness, the ability to deploy worldwide on short notice on various expeditionary platforms. Although dermatology innovation has made many therapeutics available for patients, these therapies may not be optimal for a service member. For example, injectable biologics typically require refrigeration and laboratory monitoring and increase the risk of immunosuppression, which may not be conducive to military operations. Even routine mainstay treatments, such as antibiotics for acne, may be contraindicated in a pilot. Dermatologic devices may prove nonviable if a patient is unavailable for follow-up or deploys to an area with high UV exposure. Waiting for dermatopathology diagnosis or sending out specimens can introduce further issues with deployability. Table II,^3^ for example, provides examples of the unique diagnostic and treatment considerations in treating military aviators.

**Table II:** Examples of dermatologic considerations relevant to naval aviators adapted from the Navy Aeromedical Reference and Waiver Guide

Dermatologic condition	Relevance to naval aviation	Considerations for dermatologist
Acne	- May disrupt mask seal, helmet wear, safety restraints/parachute harness	- Documentation should involve appropriate treatment for acne severity, full-body skin examination, current treatment, adverse reactions to medications, and suitability to wear flight gear
Dermatitis	- Interference with flight gear - Symptoms (eg, pruritus) can be disruptive in flight - Atopic dermatitis raises susceptibility to military irritants	- Symptoms shape disposition - Appropriate documentation as above
Psoriasis	- Relapsing nature + treatment requirements may interfere with operational responsibilities	- Certain therapies are not suitable with aviation (eg, tar products, dithranol, methotrexate, and retinoic acid) - Phototherapy (psoralen plus ultraviolet A) maintenance therapy may interrupt with operational requirements - Documentation: treatment recommendations, response to therapy

It is important to note guidance is constantly evolving.^3^

Military servicemembers are often deployed to geographically remote, austere settings that may have limited resources. Even minor issues have historically resulted in medical evacuations with significant cost and resource impacts. In response, the military health system has been developing telehealth systems far before the widespread uptake of teledermatology after COVID-19. More recently, military dermatologists have piloted incorporating synchronous teledermatoscopy to ease the characterization of skin lesions better.^4^ In settings limited by broadband connectivity, the military health system developed in 2021 the Defense Health Agency’s Global Teleconsultation Portal, an asynchronous virtual medical platform that permits asynchronous consults.^5^ These innovations lay the groundwork for modalities in civilian settings.

Service members are a global population with unique and constantly evolving health needs, requiring innovation in provider training, health care delivery, and therapies. Additional attention and scholarship can help further identify unique dermatological facets among military populations and the roles of military dermatologists.

## Conflicts of interest

None disclosed.
